# Supplementary material for: Complementarity-determining region clustering may cause CAR-T cell dysfunction
Source: Nat Commun. 2023 Aug 10;14:4732. doi: 10.1038/s41467-023-40303-z (PMC10415375; doi:10.1038/s41467-023-40303-z)
Supplement: Supplementary file 1 — Supplementary Information [file 41467_2023_40303_MOESM1_ESM.pdf]

Complementarity-determining region clustering may cause CAR-T cell dysfunction

Supplementary information

Tina Sarén[1], Giulia Saronio[1], Paula Martí Torrell[1], Xu Zhu[1], Josefin Thelander[1], Yasmin Andersson[2], Camilla Hofström[2], Marika Nestor[1], Anna Dimberg[1], Helena Persson[2], Mohanraj Ramachandran[1], Di Yu[1]*, Magnus Essand[1]*

[1] Uppsala University, Dept Immunology, Genetics, Pathology, Science for Life Laboratory, Uppsala, Sweden

[2] Royal Institute of Technology (KTH), Drug Discovery and Development Platform, Science for Life Laboratory, Solna, Sweden

* Equal contribution

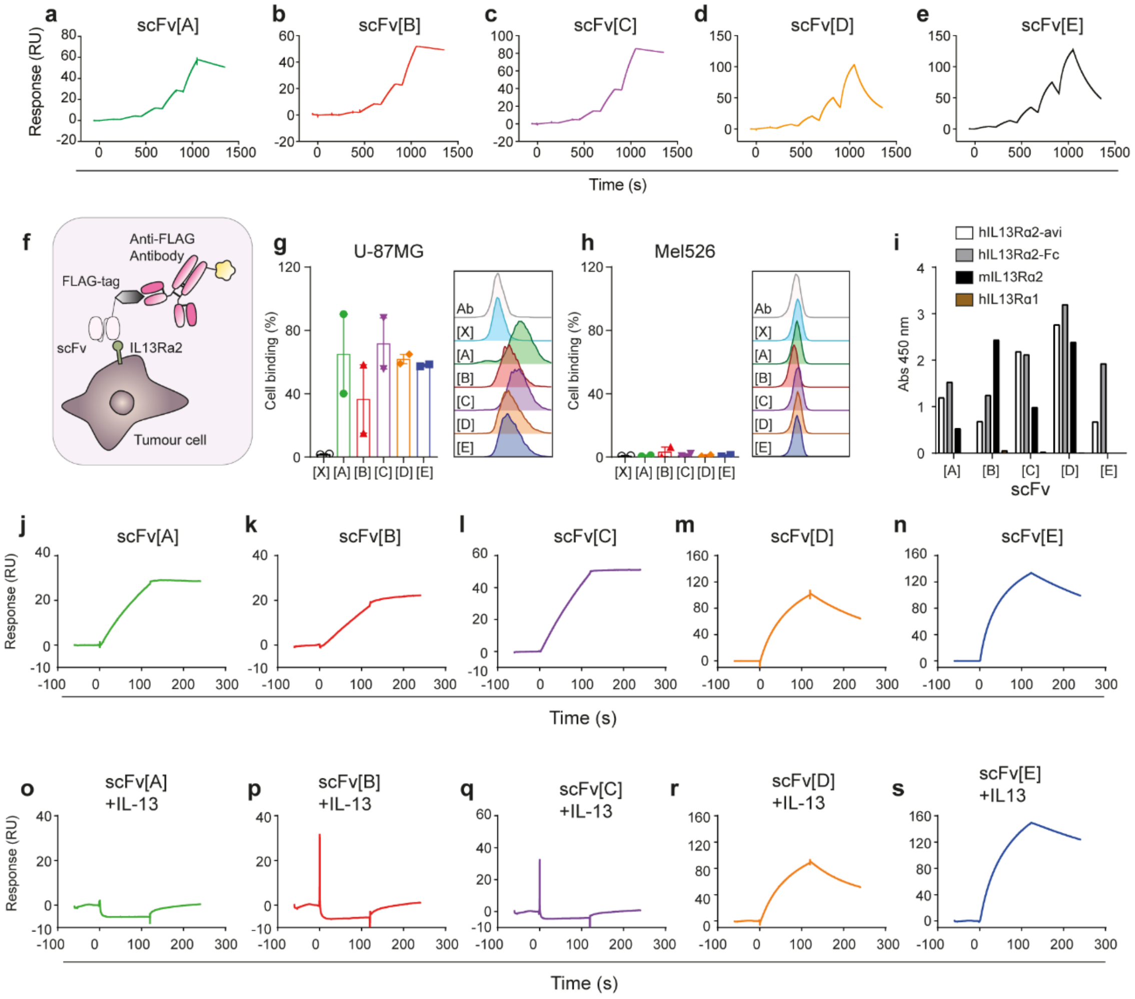

Supplementary Figure S1. Characterization of selected scFvs against IL13Rα2. (a-e) Kinetic measurement as determined by Surface Plasmon Resonance (SPR) of **a** scFv[A], **b** scFv[B], **c** scFv[C], **d** scFv[D], **e** scFv[E] against human IL13Ra2 (hIL13Rα2), using a single-cycle approach. **f** Illustration of scFv cell binding assay. Binding of scFv to **g** U-87MG glioblastoma or the **h** negative control Mel526 melanoma cell lines, and the representative histogram showing binding intensity. Irrelevant scFv[X] and only anti-FLAG antibody (Ab) were used as controls. Each dot represents one experimental replicate (n=2). Data is presented as bars of mean ± SEM**. (g, h)** Empty circle: [X], green circle: [A]; red triangle: [B]; purple reverse triangle: [C]; orange diamond: [D]; blue square: [E]. **i** The binding of scFv[A]-[E] to recombinant human avidin or Fc-tagged IL13Rα2 (hIL13Rα2-avi, hIL13Rα2-Fc), murine IL13Rα2 (mIL13Rα2), and human IL13Rα1 (hIL13Rα1). **(j-s)** Binding of the scFvs to hIL13Rα2 in the presence or absence of the ligand (IL-13) measured by SPR. Source data are provided as Source Data file.

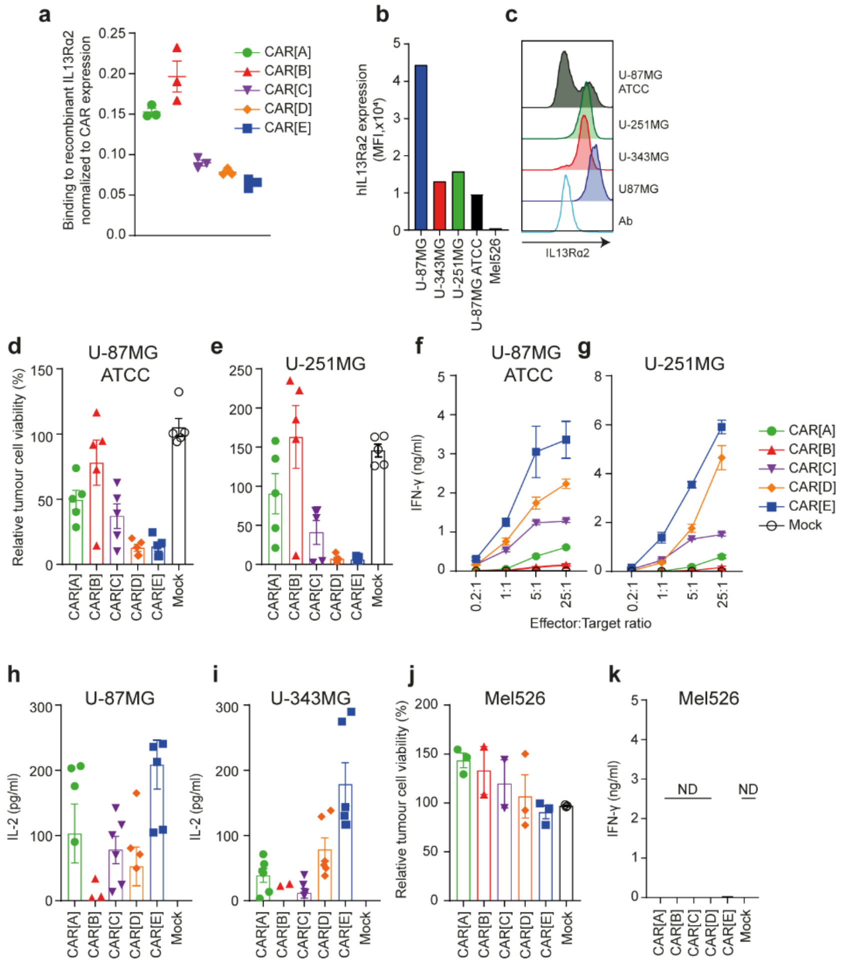

Supplementary Figure S2. CAR-Ts specifically kill IL13Rα2-expressing glioblastoma cells. a Binding of CARs, expressed in transduced Jurkat cells (defined as GFP⁺) to recombinant IL13Rα2 normalized to CAR expression level (the peak CAR value). Each dot was generated from an individual experiment (n=3) and data is presented as mean ± SEM. **(b, c)** The expression level of human IL13Rα2 (hIL13Rα2) on glioblastoma cell lines (U-87MG, U-343MG, U-251MG and U-87MG-ATCC), and absent expression on the melanoma cell line (Mel526). **c** Representative histogram showing IL13Rα2 expression on glioblastoma cell lines. (**d, e**) Relative viability of luciferase-expressing **d** U-87MG ATCC and **e** U-251MG cells after 4 days of co-culture with CAR-Ts (25:1 Effector (E) : Target (T) cell ratio). Data was generated with T cells isolated from healthy donors (n=5) and data is presented as bars of mean ± SEM. (**f, g**) IFN-γ secretion from CAR-Ts after 4d of co-culture with **f** U-87MG ATCC or **g** U-251MG. Mock-T was used as negative control. Data was generated with T cells isolated from healthy donors (**f**: n=6, **g**: n=4) and data is presented mean ± SEM. (**h, i**) IL-2 secretion from CAR-Ts when co-cultured (5:1 E:T) with **h** U-87MG and **i** U-343MG. Data was generated with T cells isolated from healthy donors (n=6) and data is presented as bars of mean ± SEM. **j** Relative viability of tumour cells and **k** IFN-γ secretion after 4d co-culture of CAR-Ts and luciferase-expressing Mel526 cells at a 25:1 (E:T) ratio. Data was generated with T cells isolated from healthy donors (**j:** CAR[A], CAR[D], CAR[E], Mock-T: n=3, CAR[B], CAR[C]: n=2; **k:** n=6) and data is presented as bars of mean ± SEM. Experiments shown in figures D-K were performed after rapid expansion on day 20 after T cell transduction. ND: Not Detected. For all graphs in this figure: green circle: CAR[A]; red triangle: CAR[B]; purple reverse triangle: CAR[C]; orange diamond: CAR[D]; blue square: CAR[E]; empty circle: Mock. Source data are provided as Source Data file. ND: not detected.

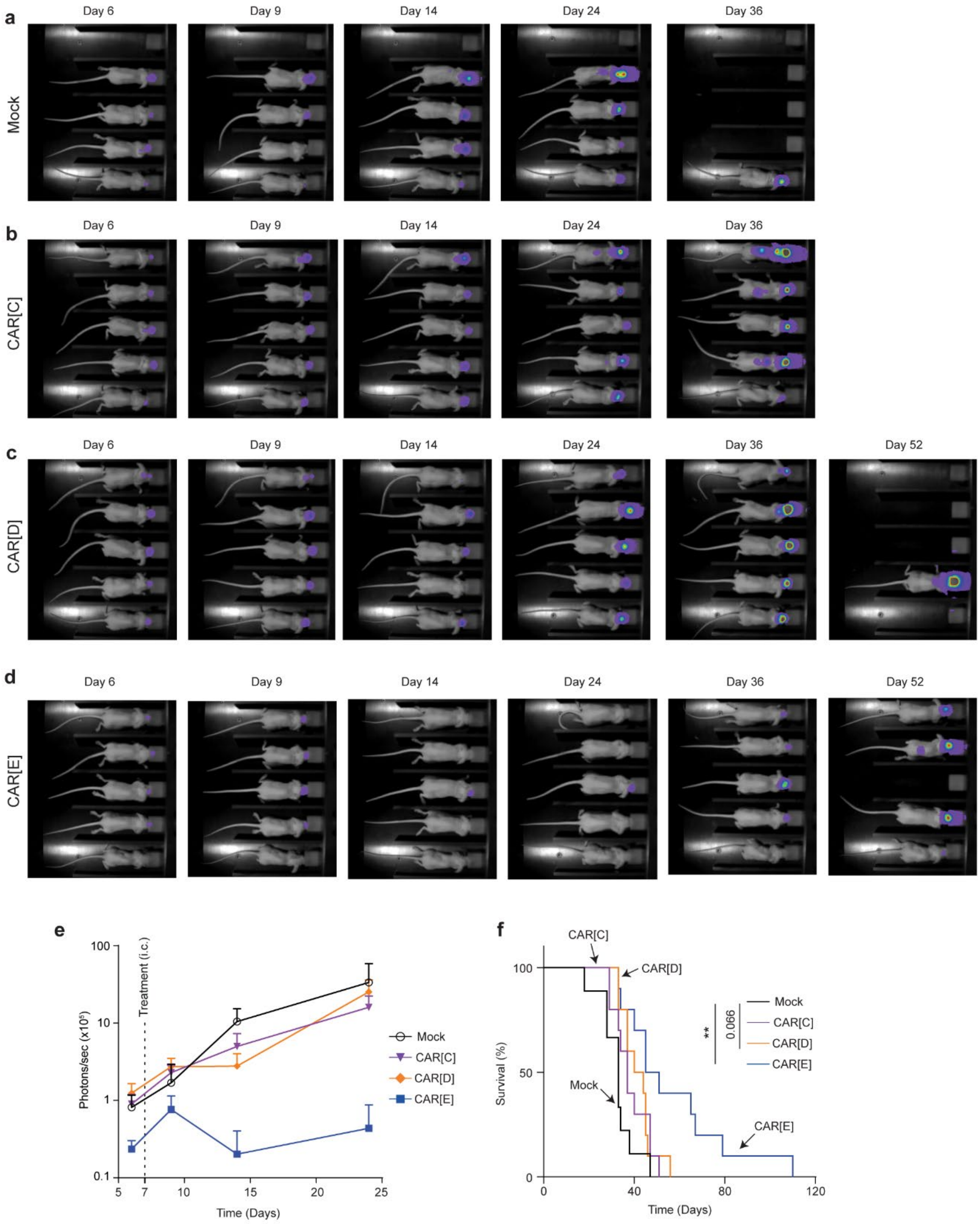

Supplementary Figure S3. Efficacy of CAR-T treatment against orthotopic U-343MG. Representative images showing luminescence signals from IVIS imaging of **a** Mock-T, **b** CAR[C]-T, **c** CAR[D]-T and **d** CAR[E]-T treated mice with orthotopic U-343MG from one representative experiment (Mock-T: n=4; CAR-T: n=5). **e** Mean tumour growth (photons/s) determined by IVIS imaging is shown from one representative experiment (Mock-T: n=4; CAR-Ts: n=5). Data is presented as mean ± SEM. Empty circle: Mock; purple reverse triangle: CAR[C]; orange diamond: CAR[D]; blue square: CAR[E]. **f** Survival data (Kaplan-Meier curve) pooled from two separate experiments (Mock-T: n=9; CAR-Ts: n=10) and curves were compared using log-rank test (**:$p \leq 0.01$). p($_{\text{CAR[D] vs. Mock}}$) = 0.066, p($_{\text{CAR[D] vs. Mock}}$) = 0.0015. Source data are provided as Source Data file.

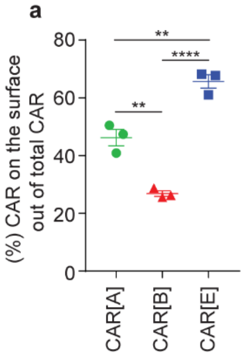

Supplementary Figure S4. CAR[A] and CAR[B] is expressed at a lower level than CAR[E] on the surface of transduced T cells. a Proportion of CAR molecules located on the T cell ($CD3^{+}GFP^{+}$) surface. p(CAR[A] vs. CAR[B]) = 0.002, p(CAR[A] vs. CAR[E]) = 0.002, p(CAR[B] vs. CAR[E]) <0.0001. Each dot represents T cells generated from one healthy donor (n=3) and data is presented as mean ± SEM. One-way ANOVA with Tukey's correction for multiple comparison was used to compare between all groups (**:p≤ 0.01, ****:p≤ 0.0001). Experiment was performed on day 13 after T cell transduction. Green circle: CAR[A]; red triangle: CAR[B], blue square: CAR[E]. Source data are provided as Source Data file.

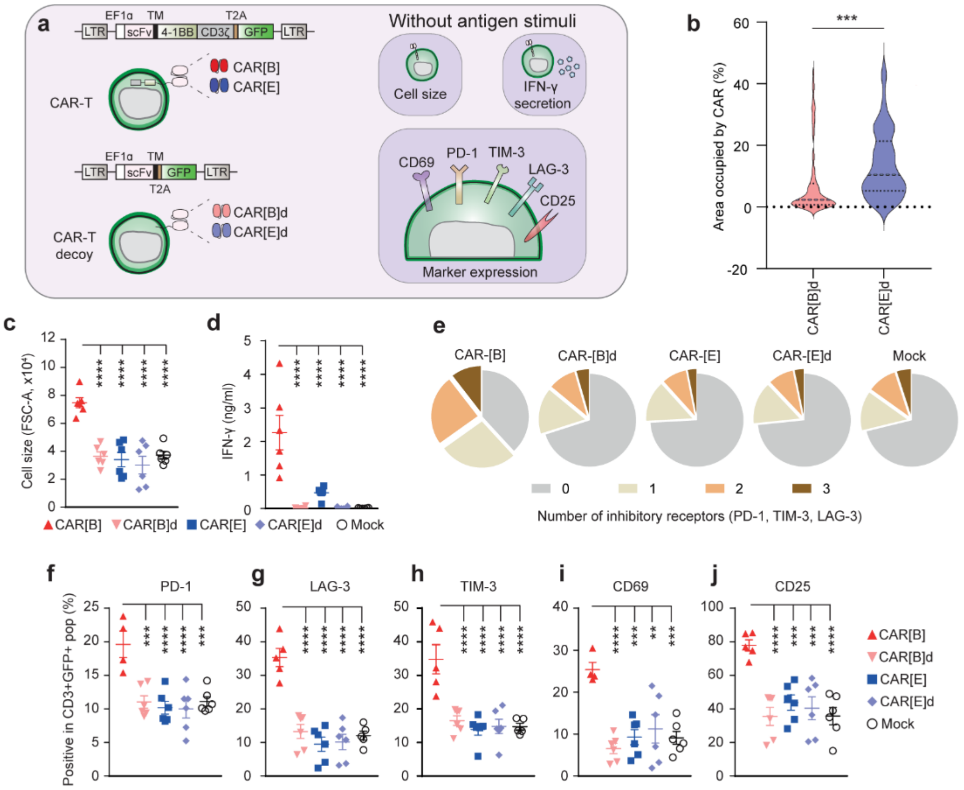

Supplementary Figure S5. Tonic signalling in CAR[B]-T is mediated through the intracellular signalling domain of the CAR. a Illustration of the experimental set-up for **b-j** wherein decoy constructs, created by removing the intracellular signalling domain, were evaluated. **b** CAR molecule distribution on the T cell surface quantified as T cell area occupied by CAR (T cells generated from 3 healthy donors). Lines represent median (dotted bold) and quartiles (dotted). Differences between groups was assessed using two-tailed unpaired t-test. $p_{(CAR[B]d\ vs.\ CAR[E]d)} = 0.0004$. Experiment was performed 5 days after T cell transduction. **c** Cell size (FSC-A) of transduced ($CD3^{+}GFP^{+}$) but unstimulated T cells, and **d** IFN-γ secretion from unstimulated cells. **(c-d)** $p_{(CAR[B]\ vs.\ All)} < 0.0001$. Each dot represents T cells isolated from one healthy donor (n=6) and data is presented as mean ± SEM. Experiments were performed 7 days after T cell transduction. **e** Pie chart displaying the proportion of $CD3^{+}GFP^{+}$ cells expressing 0, 1, 2 or 3 inhibitory receptors (PD-1, TIM-3 and LAG-3). Data is shown as mean from T cells generated from healthy donors (CAR[B]d, CAR[E], Mock-T: n=6; CAR[B]: n=5). The percentage of cells expressing **f** PD-1, **g** LAG-3, **h** TIM-3, **i** CD69 and **j** CD25 assessed 6-7 days after T cell transduction. **f** $p_{(CAR[B]\ vs.\ CAR[B]d)} = 0.0002$, $p_{(CAR[B]\ vs.\ CAR[E])} < 0.0001$, $p_{(CAR[B]\ vs.\ CAR[E]d)} < 0.0001$, $p_{(CAR[B]\ vs.\ CAR[B]d)} = 0.0003$. **g** $p_{(CAR[B]\ vs.\ All)} < 0.0001$ **h** $p_{(CAR[B]\ vs.\ All)} < 0.0001$ **i** $p_{(CAR[B]\ vs.\ CAR[B]d)} < 0.0001$, $p_{(CAR[B]\ vs.\ CAR[E])} = 0.0002$, $p_{(CAR[B]\ vs.\ CAR[E]d)} = 0.001$, $p_{(CAR[B]\ vs.\ Mock)} = 0.0002$. **j** $p_{(CAR[B]\ vs.\ CAR[B]d)} < 0.0001$, $p_{(CAR[B]\ vs.\ CAR[E])} = 0.0007$, $p_{(CAR[B]\ vs.\ CAR[E]d)} = 0.0002$, $p_{(CAR[B]\ vs.\ Mock)} < 0.0001$. Each dot represents T cells isolated from one healthy donor (**f, i**: CAR[B]d, CAR[E], CAR[E]d, Mock-T: n=6, CAR[B] n=4; **g, h**: CAR[B] n=5, CAR[B]d, CAR[E], CAR[E]d: n=6, Mock-T: n=5; **j**: CAR[B] n=5, CAR[B]d, CAR[E], CAR[E]d, Mock-T: n=6) and data is presented as mean ± SEM. Experiments were performed on days 7-9 after T cell transduction. Antibodies used for staining can be found in Supplementary Table 1. One-way ANOVA with Dunnett's correction for multiple comparison was used to compare between selected groups (*:p≤0.05, **:p≤ 0.01, ***:p≤ 0.001, ****:p≤ 0.0001). All experiments in the figure were performed 6-8 days after T cell transduction. For all graphs in this figure: red triangle: CAR[B]; light red reverse triangle: CAR[B]d; blue square: CAR[E]; light blue diamond: CAR[E]d; empty circle: Mock. EF1α: elongation factor 1 alpha, TM: transmembrane domain, T2A: self-cleaving peptide. Source data are provided as Source Data file.

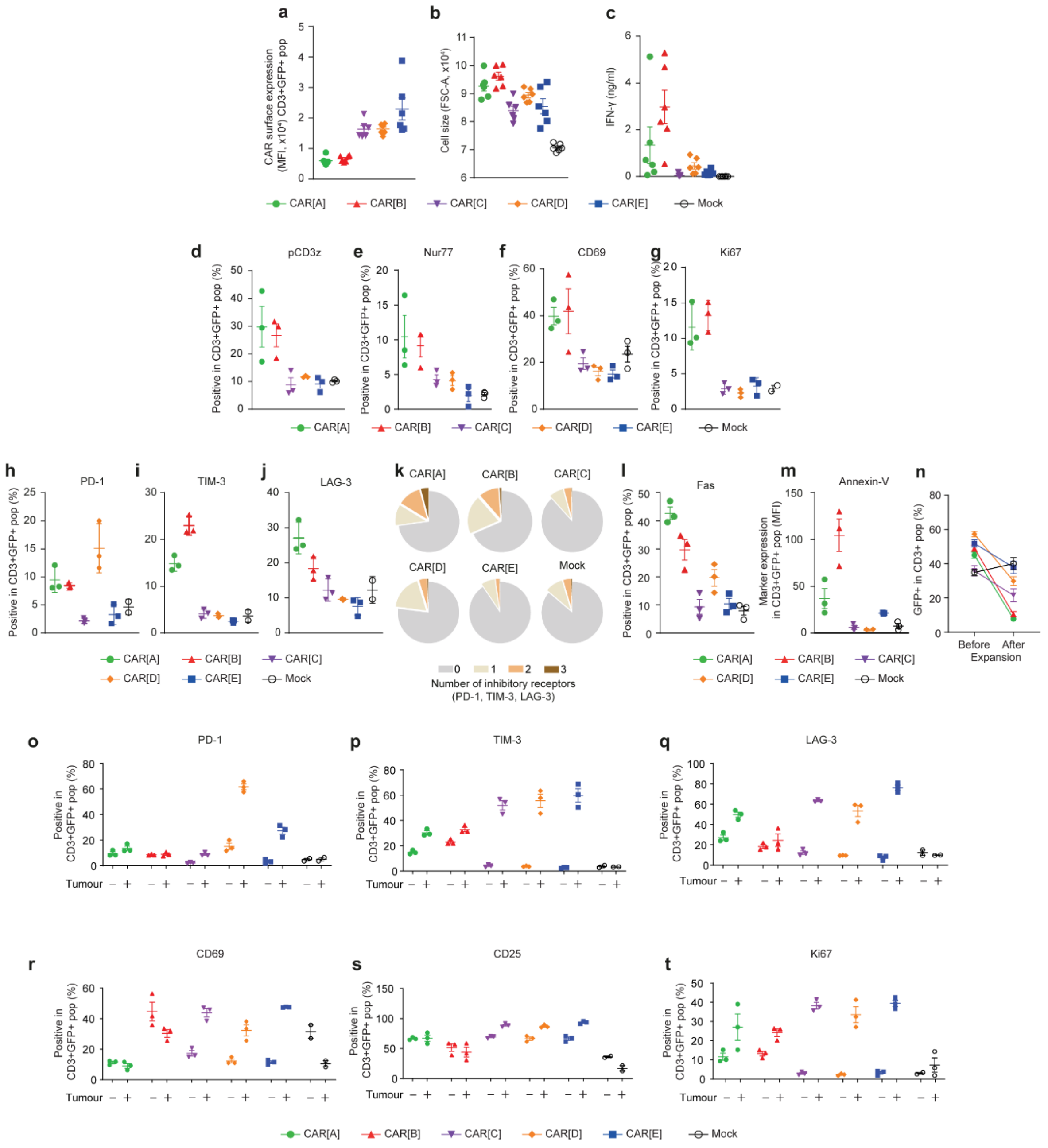

Supplementary Figure S6. CAR-Ts with antigen-independent CAR clustering respond poorly to antigen stimuli. *Please note that data from CAR[A]-T, CAR[B]-T, CAR[E]-T and Mock-T are included in Figure 2 but are also included here for comparison.* **a** CAR surface expression on CAR-Ts (defined as $CD3^{+}GFP^{+}$) 3 days after T cell transduction. **b** The cell size (FSC-A) of the CAR-Ts and Mock-T (gated as $CD3^{+}GFP^{+}$) without any antigen stimuli. **c** IFN-γ secreted overnight from rested and unstimulated CAR-Ts growth in culture medium without cytokines. Each dot represents T cells isolated from one healthy donor (n=6) and data is presented as mean ± SEM. Experiments were performed 5-7 days after T cell transduction. The expression level of **d** phosphorylated (p)CD3z, **e** Nur77, **f** CD69, **g** Ki67, **h** PD-1, **i** TIM-3, **j** LAG-3 in unstimulated CAR-Ts ($CD3^{+}GFP^{+}$). Each dot represents T cells isolated from one healthy donor (**d-f**: CAR-Ts: n=3, Mock-T: n=3; **g-j**: CAR-Ts: n=3, Mock-T: n=2) and data is presented as mean ± SEM. Experiments were performed after rapid expansion, on day 23 after T cell transduction. **k** Proportion of CAR-Ts ($CD3^{+}GFP^{+}$) expressing 0, 1, 2 or 3 inhibitory receptors (PD-1, TIM-3 or LAG-3). Data was generated using T cells isolated from healthy donors

(CAR-Ts: n=3, Mock-T: n=2) and is shown as mean. Experiment was performed after rapid expansion, on day 23 after T cell transduction. **l** Expression of Fas (%) and **m** Annexin-V (MFI) in CAR-Ts. Each dot represents T cells isolated from one healthy donor (n=3) and data is presented as mean ± SEM. **n** Reduction of CAR-Ts in culture, before and after expansion. Mock-T was used as control. Data was generated using T cells isolated from healthy donors (before n=6; after n=4) and mean is presented ± SEM. (**o-t**) Expression level of **o** PD-1, **p** TIM-3, **q** LAG-3, **r** CD69, **s** CD25 and **t** Ki67 on CAR-Ts that were left either untreated (-) or co-cultured (+) with U-87MG cells overnight. Each dot represents T cells isolated from one healthy donor (**No tumour o-t**: CAR-Ts: n=3, Mock-T: n=2; **Tumour o-s**: CAR-Ts: n=3, Mock-T: n=2; **Tumour t**: Mock-T: n=3) and data is presented as mean ± SEM. MFI: Mean fluorescence intensity. Experiment was performed after rapid expansion, on day 29 after T cell transduction. For all graphs in this figure: green circle: CAR[A]; red triangle: CAR[B]; purple reverse triangle: CAR[C]; orange diamond: CAR[D]; blue square: CAR[E]; empty circle: Mock. Source data are provided as Source Data file.

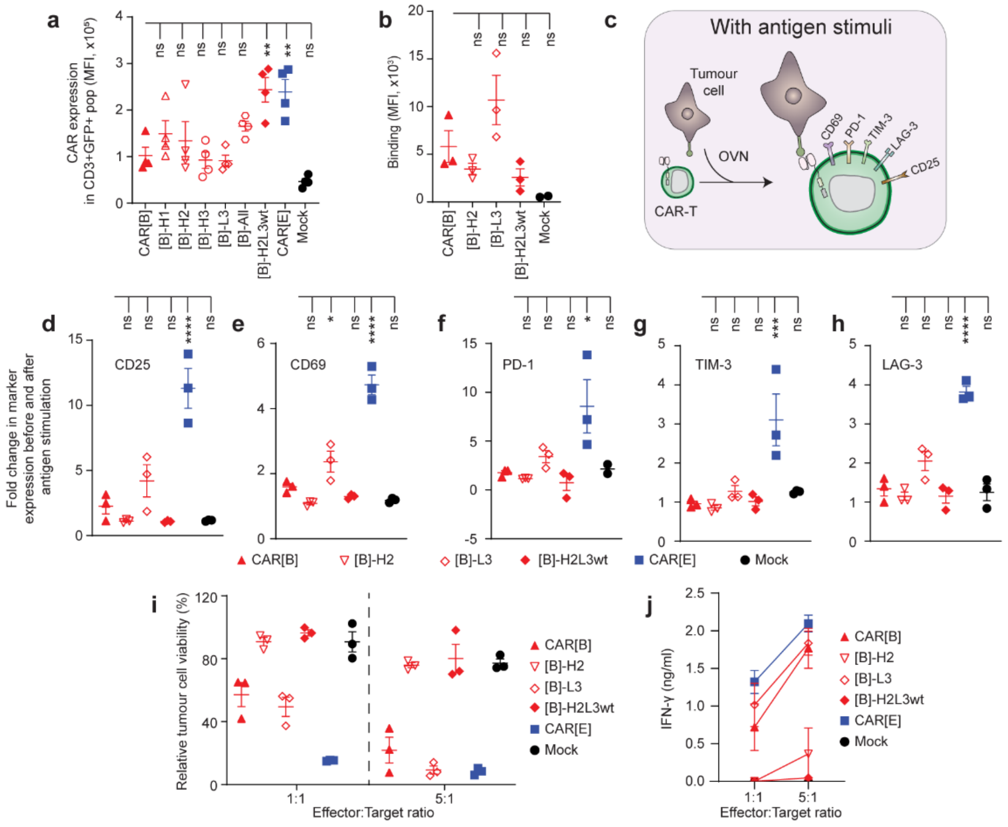

Supplementary Figure S7. CAR[B]-L3 T cells respond slightly better to antigen stimulation compared to CAR[B]-T cells. a CAR surface expression of transduced T cells (defined as $CD3^+GFP^+$ T cells). p(CAR[B] vs. [B]-H2L3wt) = 0.0012, p(CAR[B] vs. CAR[E]) = 0.002. Each dot represents T cells isolated from one healthy donor (n=4) and data is presented as mean ± SEM. Experiment was performed 5 days after T cell transduction. (**:p≤ 0.01). **b** Binding of CAR[B] and CAR[B] mutants, expressed on Jurkat cells (defined as GFP^+), to recombinant IL13Rα2. Each dot represents one individual experiment (CAR-Ts: n=3; Mock-T: n=2) and data is presented as mean ± SEM. One-way ANOVA with Dunnett's correction for multiple comparison was used to compare between selected groups. **c** Illustration of the experimental set-up to evaluate marker expression in antigen-stimulated CAR-Ts. Experiments were performed 7 days after T cell transduction. **(d-h)** Fold change in marker expression (MFI) of transduced (defined as $CD3^+GFP^+$ cells) T cells after antigen-stimulation in comparison to unstimulated cells. Fold change in expression of **d** CD25, **e** CD69, **f** PD-1, **g** TIM-3 and **h** LAG-3 after antigen-stimulation. **d** p(CAR[B] vs. CAR[E]) <0.0001, **e** p(CAR[B] vs. [B]-L3) = 0.05, p(CAR[B] vs. CAR[E]) <0.0001. **f** p(CAR[B] vs. CAR[E]) = 0.011. **g** p(CAR[B] vs. CAR[E]) = 0.0008. **h** p(CAR[B] vs. CAR[E]) <0.0001. Each dot represents T cells isolated from one healthy donor (**d, e, g, h**: n=3; **f**: CAR-Ts: n=3, Mock-T: n=2) and data is presented as mean ± SEM. Experiments were performed 7 days after T cell transduction. Antibodies used for staining can be found in Supplementary Table S1. One-way ANOVA with Dunnett's correction for multiple comparison was used to compare between selected groups (*:p≤0.05, ***:p≤ 0.001, ****:p≤ 0.0001). **i** Relative viability of U-87MG cells after 3d co-culture (1:1 and 5:1 Effector:Target cell ratio). T cells were isolated from 3 healthy donors and data is presented as mean ± SEM. **j** IFN-γ levels from the co-culture supernatant. Each dot represents the mean of T cells isolated from healthy donors (n=3) and data is presented as mean ± SEM. Experiment was performed 7 days after T cell transduction. For all graphs in this figure: red triangle: CAR[B]; empty triangle: [B]-H1; empty reverse triangle: [B]-H2; empty circle: [B]-H3, empty diamond: [B]-L3; empty hexagon: [B]-All, red diamond: [B]H2L3wt, blue square: CAR[E]; black circle: Mock. OVN: overnight. Source data are provided as Source Data file

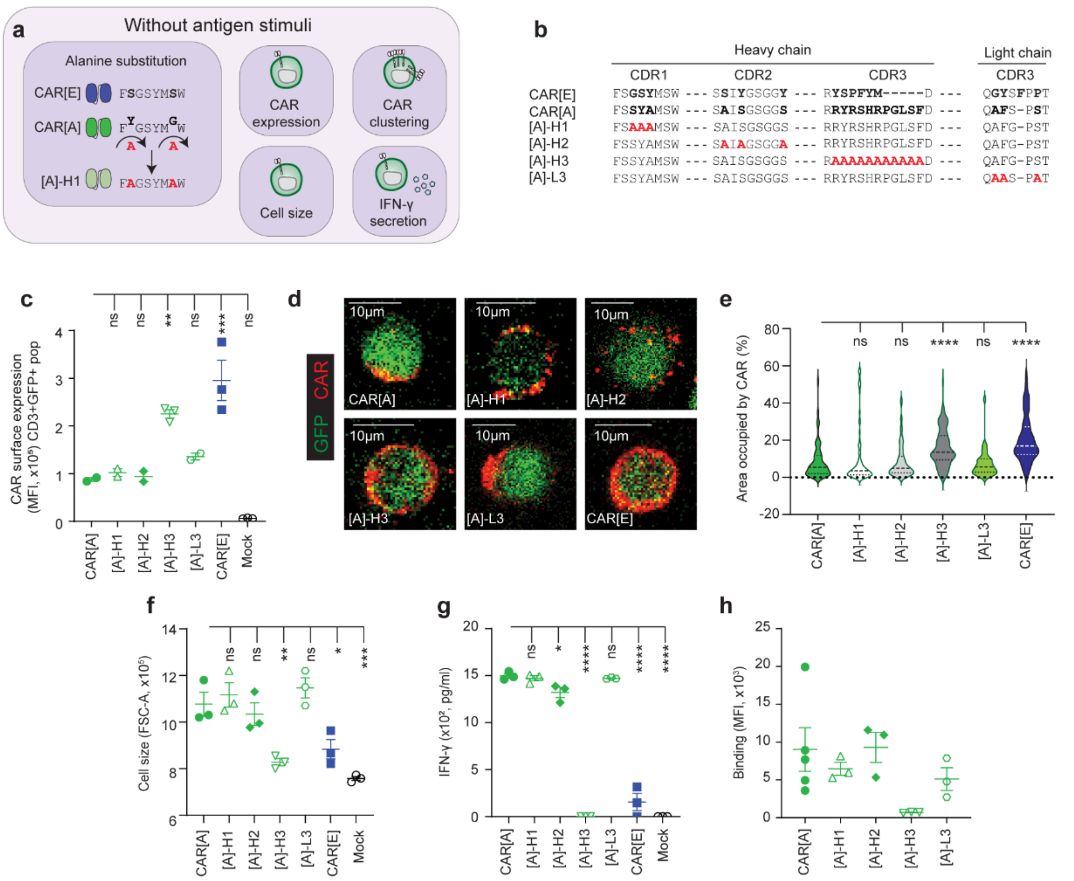

Supplementary Figure S8. Complementarity-determining region (CDR)-3 of the heavy chain mediate CAR[A] clustering and subsequent antigen-independent activation of CAR[A]-T. a Illustration of alanine substitutions and subsequent assays. **b** The variable amino acids (black bold) between CAR[E] and CAR[A] in CDR regions, and the corresponding constructs after alanine substitution (red bold). (H=Heavy chain, L= Light chain). **c** CAR surface expression of transduced T cells (defined as CD3^{+}GFP^{+} T cells). p(CAR[A] vs. [A]-H3) = 0.004, p(CAR[A] vs. CAR[E]) = 0.0001. Each dot represents T cells isolated from one healthy donor (CAR[A], [A]mutants, Mock-T: n=2, CAR[E]: n=3) and data is presented as mean ± SEM. Experiment was performed 5 days after T cell transduction. **d** Representative images showing CAR molecule distribution on the T cell surface which was **e** quantified as T cell area occupied by CAR (T cells generated from 3 healthy donors). Lines represent median (dotted bold) and quartiles (dotted). p(CAR[A] vs. [A]-H3) <0.0001, p(CAR[A] vs. CAR[E]) <0.0001. Experiment was performed 5 days after T cell transduction. **f** Cell size (FSC-A) of transduced (CD3^{+}GFP^{+}) but unstimulated T cells, and **g** IFN-γ secretion from unstimulated cells. **f** p(CAR[A] vs. [A]-H3) = 0.004, p(CAR[A] vs. CAR[E]) = 0.02, p(CAR[A] vs. Mock) = 0.0004. **g** p(CAR[A] vs. [A]-H2) = 0.049, p(CAR[A] vs. [A]-H3) <0.0001, p(CAR[A] vs. CAR[E]) <0.0001, p(CAR[A] vs. Mock) <0.0001. Each dot represents T cells isolated from one healthy donor (n=3) and data is presented as mean ± SEM. **h** Binding of transduced Jurkat cells (defined as GFP^{+}) to recombinant IL13Rα2. Each dot represents one individual experiment (CAR[A]: n=5, [A]mutants: n=3) and data is presented as mean ± SEM. One-way ANOVA with Dunnett's correction for multiple comparison was used to compare between selected groups (*:p≤0.05, **:p≤ 0.01, ***:p≤ 0.001, ****:p≤ 0.0001). MFI: Mean fluorescence intensity. For all graphs in this figure: green circle: CAR[A]; empty triangle: [A]-H1; green diamond: [A]-H2; empty reverse triangle: [A]-H3; empty hexagon: [A]-L3; blue square: CAR[E]; empty circle: Mock. Source data are provided as Source Data file.

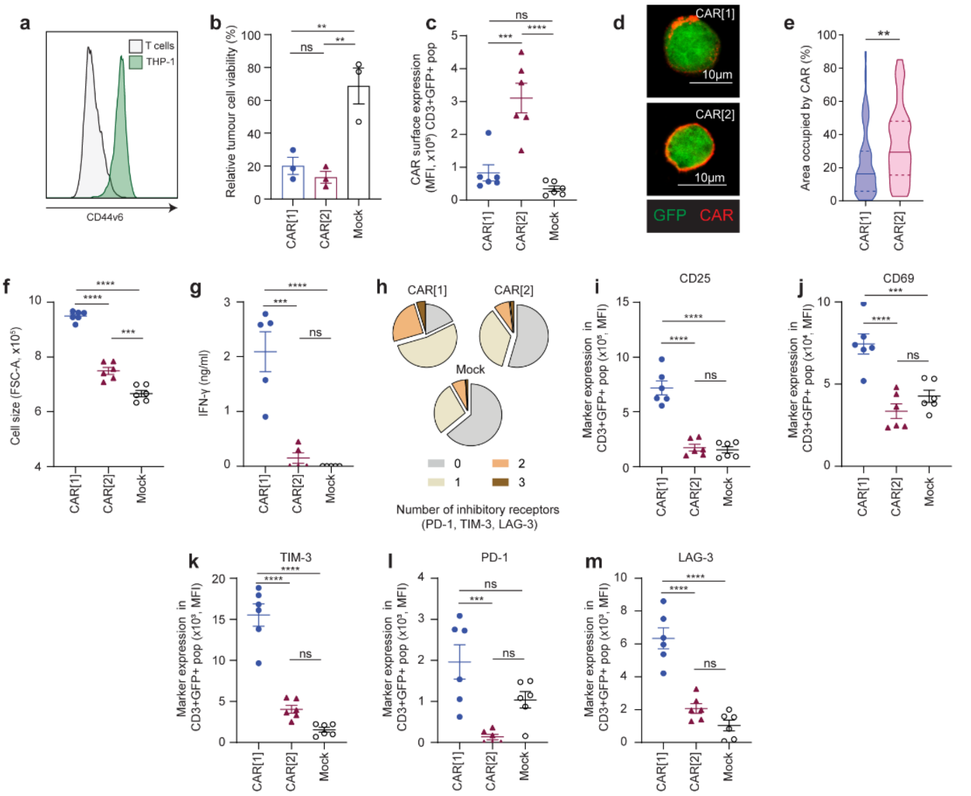

Supplementary Figure S9. CD44v6-targeted CAR[1]-T but not CAR[2]-T display antigen-independent tonic signalling. a Representative histogram showing CD44v6 expression on THP-1 cells. Stained T cells were used as negative control. **b** Relative viability of luciferase-expressing THP-1 after 4d of co-culture with CAR-Ts (3:1 Effector:Target cell ratio). Data was generated with T cells isolated from healthy donors (n=3) and data is presented as bars of mean ± SEM. **b** $p_{(CAR[1] \text{ vs. } Mock)} = 0.0079$, $p_{(CAR[2] \text{ vs. } Mock)} = 0.0041$. **C)** CAR surface expression on CAR-Ts (defined as $CD3^{+}GFP^{+}$) 5 days after T cell transduction. Each dot represents T cells isolated from one healthy donor (n=6) and data is presented as mean ± SEM. **c** $p_{(CAR[1] \text{ vs. } CAR[2])} = 0.0002$, $p_{(CAR[2] \text{ vs. } Mock)} < 0.0001$. **d** Representative images showing CAR molecule distribution on the T cell surface which was **e** quantified as T cell area occupied by CAR (T cells generated from 3 healthy donors). Lines represent median (solid) and quartiles (dotted). Differences between groups was assessed using two-tailed unpaired t-test. $p_{(CAR[1] \text{ vs. } CAR[2])} = 0.0016$. Experiment was performed 5 days after T cell transduction. **f** Cell size (FSC-A) of transduced ($CD3^{+}GFP^{+}$) but unstimulated T cells, and **g** IFN-γ secretion from unstimulated CAR-T cells. **f** $p_{(CAR[1] \text{ vs. } CAR[2])} < 0.0001$, $p_{(CAR[1] \text{ vs. } Mock)} < 0.0001$, $p_{(CAR[2] \text{ vs. } Mock)} = 0.0002$. **g** $p_{(CAR[1] \text{ vs. } CAR[2])} = 0.0001$, $p_{(CAR[1] \text{ vs. } Mock)} < 0.0001$. Each dot represents T cells isolated from one healthy donor (**f**: n=6; **g**: n=5) and data is presented as mean ± SEM. **h** Proportion of CAR-Ts ($CD3^{+}GFP^{+}$) expressing either 1, 2 or 3 inhibitory receptors (PD-1, TIM-3 and LAG-3). Data was generated using T cells isolated from healthy donors (n=3) and is shown as mean. The proportion of $CD3^{+}GFP^{+}$ cells expressing **i** CD25, **j** CD69, **k** TIM-3, **l** PD-1, **m** LAG-3**. i** $p_{(CAR[1] \text{ vs. } CAR[2])} < 0.0001$, $p_{(CAR[1] \text{ vs. } Mock)} < 0.0001$. **j** $p_{(CAR[1] \text{ vs. } CAR[2])} < 0.0001$, $p_{(CAR[1] \text{ vs. } Mock)} = 0.0009$. **k** $p_{(CAR[1] \text{ vs. } CAR[2])} < 0.0001$, $p_{(CAR[1] \text{ vs. } Mock)} < 0.0001$. **l** $p_{(CAR[1] \text{ vs. } CAR[2])} = 0.0007$. **m** $p_{(CAR[1] \text{ vs. } CAR[2])} < 0.0001$, $p_{(CAR[1] \text{ vs. } Mock)} < 0.0001$. Each dot represents T cells isolated from one healthy donor (n=6) and data is presented as mean ± SEM. One-way ANOVA with Tukey's correction for multiple comparison was used to compare between groups (**:$p \leq 0.01$, ***:$p \leq 0.001$, ****:$p \leq 0.0001$). MFI: Mean fluorescence intensity. For all graphs in this figure: blue circle: CAR[1]; dark red triangle: CAR[2]; empty circle: Mock. Source data are provided as Source Data file.

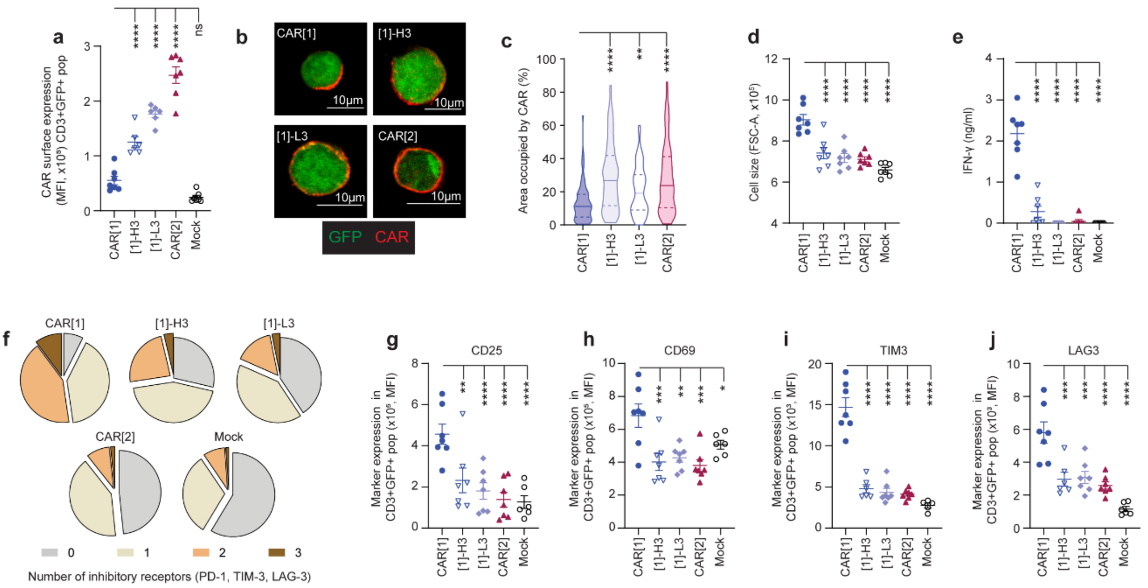

Supplementary Figure S10. Complementarity-determining region (CDR)-3 of the heavy chain and light chain mediate CAR[1] clustering and subsequent antigen-independent activation of CD44v6-directed CAR[1]-T. a CAR surface expression on CAR-Ts (defined as $CD3^+GFP^+$) 5 days after T cell transduction. p(CAR[1] vs. [1]-H3) <0.0001, p(CAR[1] vs. [1]-L3) <0.0001, p(CAR[1] vs. CAR[2]) <0.0001. Each dot represents T cells isolated from one healthy donor (CAR[1], CAR[2], Mock-T: n=7; [1]-H3, [1]-L3: n=6) and data is presented as mean ± SEM. **b** Representative images showing CAR molecule distribution on the T cell surface which was **c** quantified as T cell area occupied by CAR (T cells generated from 3 healthy donors). Lines represent median (solid) and quartiles (dotted). p(CAR[1] vs. [1]-L3) = 0.0077, p(CAR[1] vs. [1]-H3) <0.0001, p(CAR[1] vs. CAR[2]) <0.0001. Experiments were performed 5 days after T cell transduction. **d** Cell size (FSC-A) of transduced ($CD3^+GFP^+$) but unstimulated T cells, and **E)** IFN-γ secretion from unstimulated cells. **(d-e)** p(CAR[1] vs. All) <0.0001. Each dot represents T cells isolated from one healthy donor (n=7) and data is presented as mean ± SEM. **f** Proportion of CAR-Ts ($CD3^+GFP^+$) expressing either 1, 2 or 3 inhibitory receptors (PD-1, TIM-3 and LAG-3) (Mock-T: n=4; CAR-Ts: n=4). The proportion of $CD3^+GFP^+$ cells expressing **g** CD25, **h** CD69, **i** TIM-3, **j** LAG-3. **g** p(CAR[1] vs. [1]-H3) = 0.0047, p(CAR[1] vs. [1]-L3) = 0.0006, p(CAR[1] vs. CAR[2]) <0.0001, p(CAR[1] vs. CAR[2]) <0.0001. **h** p(CAR[1] vs. [1]-H3) = 0.0006, p(CAR[1] vs. [1]-L3) = 0.0017, p(CAR[1] vs. CAR[2]) = 0.0002, p(CAR[1] vs. Mock) = 0.046. **i** p(CAR[1] vs. All) <0.0001, **j** p(CAR[1] vs. [1]-H3) = 0.0002, p(CAR[1] vs. [1]-L3) = 0.0002, p(CAR[1] vs. CAR[2]) <0.0001, p(CAR[1] vs. Mock) <0.0001. Each dot represents T cells isolated from one healthy donor (**g, h:** CAR-Ts: n=7; Mock-T: n=6; **i:** CAR[1], CAR[2], [1]-L3: n=7; [1]-H3: n=6; Mock-T: n=5; **j:** CAR[1], CAR[2], [1]-L3: n=7; [1]-H3, Mock-T: n=6) data is presented as mean ± SEM. One-way ANOVA with Dunnett's correction for multiple comparison was used to compare between selected groups (*:p≤0.05, **:p≤ 0.01, ***:p≤ 0.001, ****:p≤ 0.0001). MFI: Mean fluorescence intensity. For all graphs in this figure: blue circle: CAR[1]; empty triangle: [1]-H3; light blue diamond: [1]-L3; dark red triangle: CAR[2]; empty circle: Mock. Source data are provided as Source Data file.

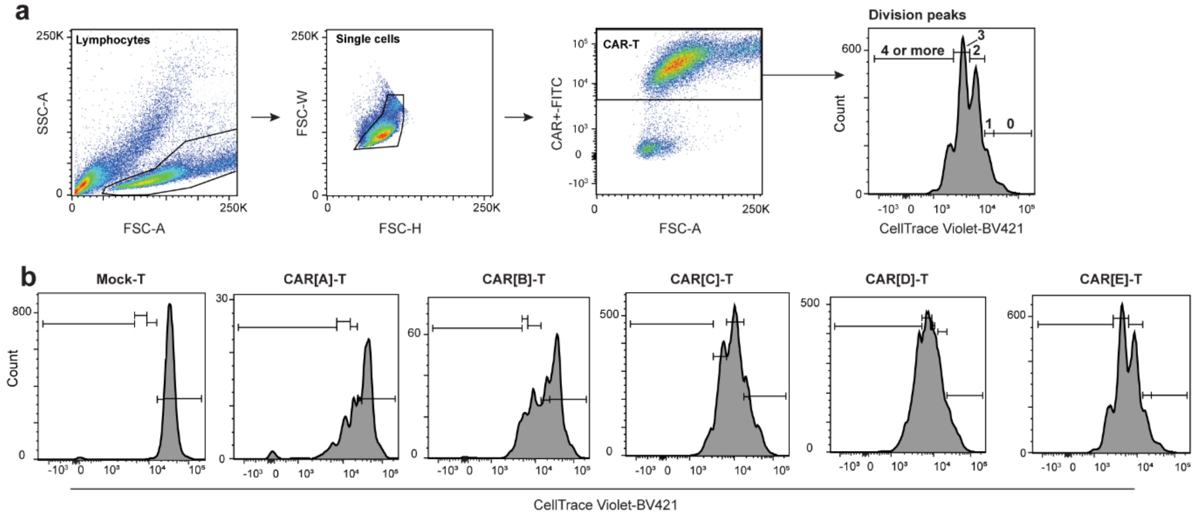

Supplementary Figure S11. Gating strategy used to assess proliferation of CAR-Ts. a Gating strategy used to identify CAR-Ts (GFP^{+}) and assess their cell division in Fig. 1I. **b** Representative histograms of division peaks from each CAR-T construct used in Fig. 1i.

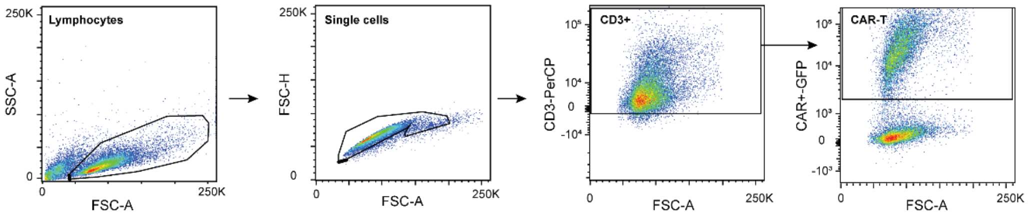

Supplementary Figure S12. Gating strategy used to identify CAR-Ts. Gating strategy used to identify CAR-Ts (defined as $CD3^{+}GFP^{+}$ population) in order to assess CAR-T cell size in: Fig. 2h, Fig. 4g, Fig. S5c, Fig. S6b, Fig. S8f, Fig. S9f, Fig. S10d and CAR expression level on CAR-Ts in: Fig. 2b, Fig. 4c-d, Fig. S6a, Fig. S7a, Fig. S8c, Fig. S9c, Fig. S10a.

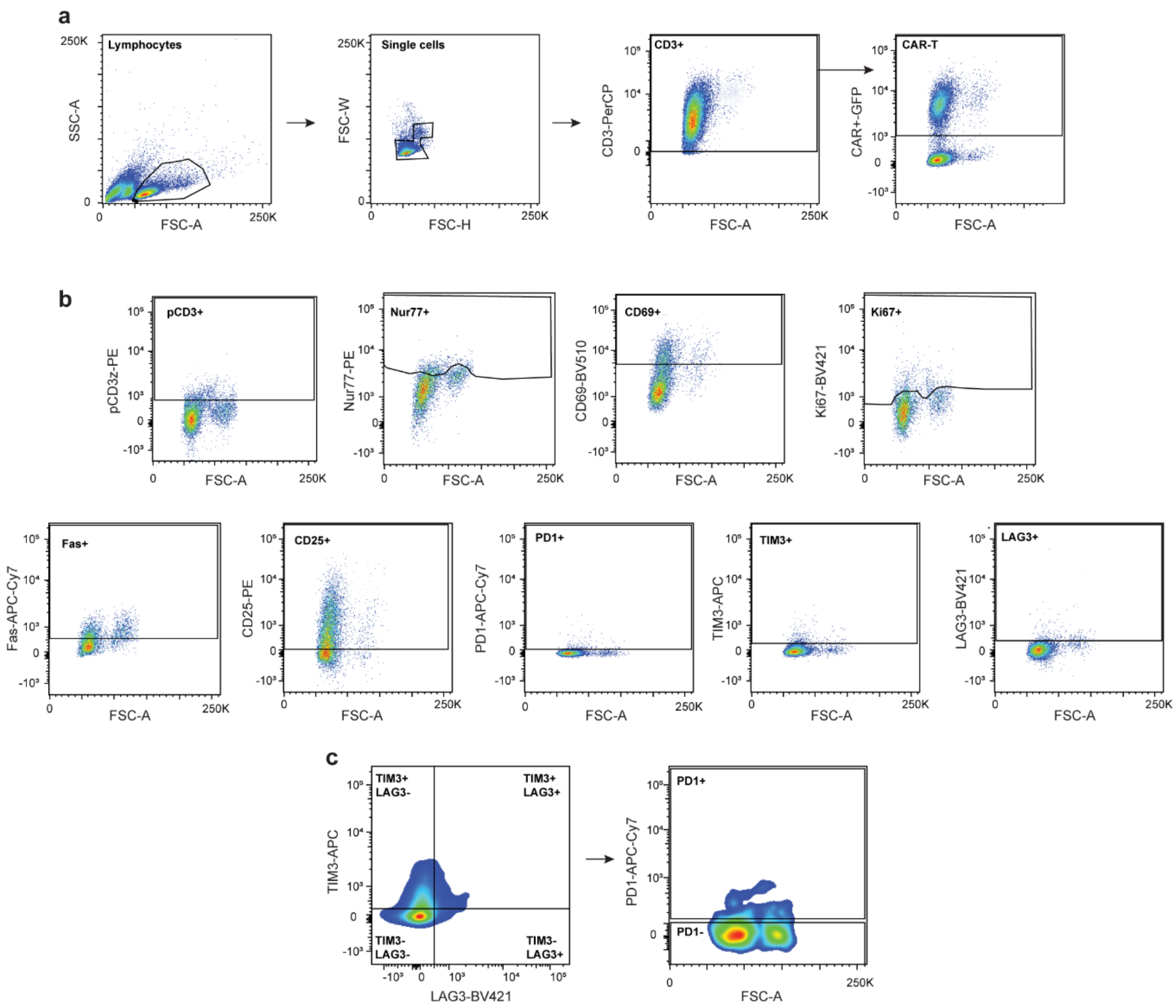

Supplementary Figure S13. Gating strategy for flow cytometry analysis of markers on CAR-Ts. a Gating strategy used to identify CAR-Ts (defined as CD3^{+}GFP^{+}). **b** Gating strategy used to determine pCD3z^{+}, Nur77^{+}, CD69^{+}, Ki67^{+}, Fas^{+}, CD25^{+}, PD1^{+}, TIM3^{+} and LAG3^{+} CAR-Ts, defined as in (**a**), in Fig. 2j-m, Fig. 2o-p, Fig. 2s-x, Fig. 4i, Fig. S5f-j, Fig. S6d-j, Fig. S6l-m, Fig. S6o-t, Fig. S7d-h and Fig. S9i-m, Fig. S10g-j. **c** Gating strategy used to identify CAR-Ts, defined as in (**a**), with 0, 1, 2 or 3 inhibitory receptors (PD-1, TIM-3 and LAG-3) in Fig. 2n, Fig.4j, Fig. S5e, Fig. S6k, Fig. S9h and Fig. S10f.

Supplementary Table S1. List of antibodies and dyes used for scFv binding assays and for flow cytometry assays.

	Marker	Clone	Fluorochrome	Dilution	Company	Identifier (Cat # and RRID)
**Used for CAR expression**	Human IgG (H+L) CAR	Polyclonal	AF647	1:100	Invitrogen	# A-21445 RRID: AB_2535862
	CD3	UCHT1	BV421	1:100	BD Bioscience	# 562426 RRID:AB_11152082
**Used for scFv-binding assays**	FLAG	M2	PE	1:500	ProZyme	# PJ315 RRID:AB_2895555
	FLAG	M2	HRP	1: 10 000	Sigma-Aldrich	# A8592 RRID:AB_439702
	FLAG	M2	-	1:20	Sigma-Aldrich	# F1804 AB_262044
**Used for CAR-T surface marker panel**	CD3	SP34-2	BB700	1:200	BD	# 566517 RRID:AB_2744378
	PD-1	EH12.2H7	APC/Cy7	1:200	BioLegend	# 329922 RRID:AB_10933429
	TIM-3	F38-2E2	APC	1:200	BioLegend	# 345012 RRID:AB_2561718
	LAG-3	T47-530	BV421	1:200	BD	# 565720 RRID:AB_2744330
	CD69	FN50	BV510	1:200	BioLegend	# 310936 RRID:AB_2563834
	CD25	BC96	PE	1:200	BioLegend	# 302606 RRID:AB_314276
	AnnexinV	-	PE/Cy7	1:20	BioLegend	# 640950
**Used for CAR-T phospho panel**	CD3	SP34-2	BB700	1:200	BD	# 566517 RRID:AB_2744378
	Human IgG (H+L) CAR	Polyclonal	AF647	1:100	Invitrogen	# A-21445 RRID: AB_2535862
	Phospho-CD3 zeta	3ZBR4S	PE	1:100	eBioScience	# 12-2478-42 RRID:AB_2744700
**Used for CAR-T intra-nuclear panel**	CD3	SP34-2	BB700	1:200	BD	# 566517 RRID:AB_2744378
	Fas	DX2	APC/Cy7	1:200	BioLegend	# 305636 RRID:AB_2566111
	Ki67	Clone Ki-67	BV421	1:200	BioLegend	# 350506 RRID:AB_2563860
	Nur77	12.14	PE	1:100	eBioScience	# 12-5965-82 RRID:AB_1257209
**Used for IL13Rα2 detection**	IL13Rα2	Polyclonal	Unconjugated	1:25	R&D	# AF146 RRID:AB_354809
	Goat IgG (H+L)	Polyclonal	AF647	1:200	Invitrogen	# A-21447 RRID:AB_141844
**Used for proliferation assay**	CD3	UCHT1	PerCP	1:200	BioLegend	# 300427 RRID:AB_893300
**Used for CD44v6 detection**	CD44v6	2F10	BV421	1:100	BD	#749713 RRID:AB_2873967

Supplementary Table S2. List of genes used as input for TFacts analysis

Genes significantly upregulated in CAR[B]-T compared to CAR[E]-T and Mock-T			Genes significantly downregulated in CAR[B]-T compared to CAR[E]-T and Mock-T
ACTN1	HSD11B1	NSD2	RORC
ACVR1B	ICAM1	OAS1	CCR6
AURKA	ICOSLG	OAS3	TRDV2
BATF	IFI30	PECAM1	MAF
BATF3	IFI6	PHGDH	CCR2
BCL2L1	IFIT1	PIK3R3	CCL20
BUB1	IFIT3	PRKCD	CCR5
CASP3	IFNGR2	PTGER4	IL1R1
CCL1	IGF1R	PTGS2	CXCR6
CCL22	IKBKE	RDH10	CMKLR1
CCL25	IKZF2	SGO2	IL17F
CCL3/L1	IKZF4	SHMT2	VSIR
CCR8	IL13	SMC2	PROCR
CD200	IL15	SPIB	MYC
CD247	IL1A	SRC	CD7
CD276	IL2	STAT1	PTGDR2
CD38	IL21	TCF7	TCL1A
CD40	IL3	TFRC	DUSP1
CD80	IL36A	TNFRSF10B	SGK3
CD84	IL6ST	TNFRSF18	CCL5
CD9	IRF4	TNFRSF4	CXCL2
CEACAM1	IRF5	TNFRSF9	RORA
CIITA	IRF6	TNFSF13B	CPT1A
CPT1B	IRF7	TOX	KLRB1
CRLF2	IRF8	UBE2F	BID
CSF2	JAG1	USP18	SELPLG
CSF2RA	LIF	XAF1	CD8B
CTNND1	LTA	XCL1/2	TIMP1
CXCL10	MAML3		IL7
CXCL13	MID1IP1		TNFRSF11A
CXCR4	MKI67		CALM1
CXCR5	MMP9		SLC2A1
EBI3	MT2A		TGFB1
ENTPD1	MTHFD2		CCR4
EOMES	MX1		CD40LG
FAM30A	NCAPG2		FOXP3
Feature ID	NCAPH		SIGLEC5
GZMB	NEK2		PPP3CA
GZMK	NFAT5		GZMM
HAVCR2	NFIL3		RPL3
HDAC7	NFKB2		PRKCB
HIF1A	NFKBIA		TRBV29-1
HLA-DRA	NME1		IL7R
HLA-DRB1	NOD2		TBX21
HMGCR	NR3C1		TRAV7
			PRF1
			CASP8
			CCL19
			MINOS1
			RPL23
			MS4A1
			LILRB3
