# Supplementary material for: Complementarity-determining region clustering may cause CAR-T cell dysfunction
Source: Nat Commun. 2023 Aug 10;14:4732. doi: 10.1038/s41467-023-40303-z (PMC10415375; doi:10.1038/s41467-023-40303-z)
Supplement: Supplementary file 3 — Description of Additional Supplementary Files [file 41467_2023_40303_MOESM3_ESM.pdf]

**Description of Additional Supplementary Files**

File Name: Supplementary Data 1
Description: The analysis for the differentially expressed genes presented in Figure 3.
