# Supplementary material for: Complementarity-determining region clustering may cause CAR-T cell dysfunction
Source: Nat Commun. 2023 Aug 10;14:4732. doi: 10.1038/s41467-023-40303-z (PMC10415375; doi:10.1038/s41467-023-40303-z)
Supplement: Supplementary file 5 — Reporting Summary [file 41467_2023_40303_MOESM5_ESM.pdf]

nature portfolio

Corresponding author(s): Magnus Essand, Di Yu

Last updated by author(s): Jul 5, 2023

Reporting Summary

Nature Portfolio wishes to improve the reproducibility of the work that we publish. This form provides structure for consistency and transparency in reporting. For further information on Nature Portfolio policies, see our Editorial Policies and the Editorial Policy Checklist.

Statistics

For all statistical analyses, confirm that the following items are present in the figure legend, table legend, main text, or Methods section.

n/a	Confirmed	
☐	☒	The exact sample size (n) for each experimental group/condition, given as a discrete number and unit of measurement
☐	☒	A statement on whether measurements were taken from distinct samples or whether the same sample was measured repeatedly
☐	☒	The statistical test(s) used AND whether they are one- or two-sided<br>*Only common tests should be described solely by name; describe more complex techniques in the Methods section.*
☐	☒	A description of all covariates tested
☐	☒	A description of any assumptions or corrections, such as tests of normality and adjustment for multiple comparisons
☐	☒	A full description of the statistical parameters including central tendency (e.g. means) or other basic estimates (e.g. regression coefficient) AND variation (e.g. standard deviation) or associated estimates of uncertainty (e.g. confidence intervals)
☐	☒	For null hypothesis testing, the test statistic (e.g. F, t, r) with confidence intervals, effect sizes, degrees of freedom and P value noted<br>*Give P values as exact values whenever suitable.*
☒	☐	For Bayesian analysis, information on the choice of priors and Markov chain Monte Carlo settings
☒	☐	For hierarchical and complex designs, identification of the appropriate level for tests and full reporting of outcomes
☒	☐	Estimates of effect sizes (e.g. Cohen's d, Pearson's r), indicating how they were calculated

Our web collection on statistics for biologists contains articles on many of the points above.

Software and code

Policy information about availability of computer code

Data collection	For flow cytometry: BD FACSDiva 8.0.2, BD FACSDiva v9.0.1, CytExpert 2.4. For microscopy: Leica Application Suite X 3.0.1.15878, Zen Blue 1.1.2.0.
Data analysis	For binding kinetics: BIAeval v.3.1. For flow cytometry: FlowJo v10.8.1. For image analysis: ImageJ v1.51, Leica Application Suite X 3.0.1.15878, For nanostring gene analysis: nSolver 4.0, Partek flow 10.0.22.0828. For statistical analysis: GraphPad Prism 9.01.

For manuscripts utilizing custom algorithms or software that are central to the research but not yet described in published literature, software must be made available to editors and reviewers. We strongly encourage code deposition in a community repository (e.g. GitHub). See the Nature Portfolio guidelines for submitting code & software for further information.

Data

Policy information about availability of data

All manuscripts must include a data availability statement. This statement should provide the following information, where applicable:

- Accession codes, unique identifiers, or web links for publicly available datasets
- A description of any restrictions on data availability
- For clinical datasets or third party data, please ensure that the statement adheres to our policy

Source data are provided with this paper.
The gene expression data generated in this study has been deposited in the Mendeley Data database under accession code: doi: 10.17632/k4r8ynxyz9.3. The access

to other pre-processed data files (e.g. flow cytometry, images, SPR etc) can be obtained by contacting the corresponding authors due to large data sets.
Sequence for extracellular domain of IL13Rα2: Uniprot Q14627 aa 29-501

Human research participants

Policy information about studies involving human research participants and Sex and Gender in Research.

Reporting on sex and gender	*Use the terms sex (biological attribute) and gender (shaped by social and cultural circumstances) carefully in order to avoid confusing both terms. Indicate if findings apply to only one sex or gender; describe whether sex and gender were considered in study design whether sex and/or gender was determined based on self-reporting or assigned and methods used. Provide in the source data disaggregated sex and gender data where this information has been collected, and consent has been obtained for sharing of individual-level data; provide overall numbers in this Reporting Summary. Please state if this information has not been collected. Report sex- and gender-based analyses where performed, justify reasons for lack of sex- and gender-based analysis.*
Population characteristics	The human buffy coats obtained from healthy blood donors had been anonymized.
Recruitment	Peripheral blood mononuclear cells were isolated by Ficoll-Paque (GE Healthcare Life Science, Uppsala, Sweden) from fresh buffy coats of healthy anonymized donors, collected at the Blood Centre at Uppsala University Hospital.
Ethics oversight	Because the samples had been anonymized, an ethical permit was not required.

Note that full information on the approval of the study protocol must also be provided in the manuscript.

Field-specific reporting

Please select the one below that is the best fit for your research. If you are not sure, read the appropriate sections before making your selection.

☒ Life sciences ☐ Behavioural & social sciences ☐ Ecological, evolutionary & environmental sciences

For a reference copy of the document with all sections, see nature.com/documents/nr-reporting-summary-flat.pdf

Life sciences study design

All studies must disclose on these points even when the disclosure is negative.

Sample size	No sample size calculation was performed to predetermine sample size in either in vivo/ex vivo or in vitro experiments. Sample size for in vivo/ex vivo experiments was determined based on previous experience with biological variation in the in vivo models used (PMID: 26363010, 26645582) and accounting for the 3R principle. The same applies to in vitro experiments, which were conducted with T cells isolated from healthy donor blood. We believe that the chosen sample size is sufficient since our results are reproducible and consistent with respect to statistical differences between the treatment groups.
Data exclusions	Data points in in-vitro experiments, where CAR-T from healthy donors could not be successfully engineered were excluded. The possible reasons are poor viability or poor expansion.
Replication	In vivo studies and in vitro assays were repeated multiple times (at least two independent experiments).<br>In vitro studies were performed with T cells isolated from at least 3 donors and assays were performed by different scientists to ensure reproducibility. T cells from donors that did not survive in vitro culturing or had poor viability were excluded from downstream analysis. The manuscript only includes the data which was reproducible.
Randomization	For in vivo studies, allocation of mice to each treatment group was random. Mice of similar ages were randomly divided in treatment groups at the start of each experiment. Since the mice were of similar age and background, further randomization was not necessary.<br>T cells isolated from each individual donor were used to generate all the different CAR-T cell constructs. Thus, it accounts for the donor variability in the in vitro assays.
Blinding	The researchers were not blinded because they needed to be aware of the risks associated with handling the different lentiviruses and donor-derived T cells.

Reporting for specific materials, systems and methods

We require information from authors about some types of materials, experimental systems and methods used in many studies. Here, indicate whether each material, system or method listed is relevant to your study. If you are not sure if a list item applies to your research, read the appropriate section before selecting a response.

Materials & experimental systems

n/a	Involved in the study	
	x	Antibodies
	x	Eukaryotic cell lines
x		Palaeontology and archaeology
	x	Animals and other organisms
x		Clinical data
x		Dual use research of concern

Methods

n/a	Involved in the study	
x		ChIP-seq
	x	Flow cytometry
x		MRI-based neuroimaging

Antibodies

Antibodies used	All antibodies used in this study are listed in Supplementary table S1. Information regarding fluorochromes, clone, dilution, catalog number and company are also reported in the above mentioned Supplementary table S1
Validation	Relevant publication or validations assays for all antibodies used for immunofluorescence staining or flow cytometry can be found on the RRID portal (https://scicrunch.org/resources) or on the manufacturer's website. For easy access to this information, the research resource identifiers (RRIDs) and catalogue numbers of all antibodies used for are provided in Supplementary table S1.<br><br>In addition:<br>- We validated the Human IgG (H+L) used for CAR detection by staining CAR-T cells along with relevant negative controls (untransduced or Mock transduced T cells) prior to performing the assays presented in this study<br>- All the antibodies used for flow cytometry were validated on unstimulated and stimulated T cells isolated from blood of healthy donors together with the relevant fluorescence minus one (FMO) controls<br>- We validated the IL13Ra2 antibody using positive (U-87MG) and negative (Mel526) cells<br>- We validated the CD44v6 antibodies positive THP-1 and negative T cells<br>- The M2 FLAG antibodies have previously been used in the scFv/antibody production pipeline (doi: 10.1074/jbc.RA120.012893) used by co-authors

Eukaryotic cell lines

Policy information about cell lines and Sex and Gender in Research

Cell line source(s)	U-87MG (UU), U-87MG (ATCC-HTB14), U-343MG (UU), U-251MG (UU), Mel526 (Cellosaurus, CVCL_8051), THP-1 cells (TIB-202, ATCC), Jurkat cells (D1.1, ATCC CRL-10915), (ATCC, CRL-1711)
Authentication	The cell lines used in this study were not authenticated.
Mycoplasma contamination	All cell lines were routinely tested and are negative for mycoplasma.
Commonly misidentified lines<br>(See ICLAC register)	In this paper both the original U-87MG Uppsala and U-87MG (ATCC) were used as they are both are likely glioblastoma cell lines and express IL13Rα2. PMID: 27582061 Allen M, Bjerke M, Edlund H, Nelander S, Westermark B. Origin of the U87MG glioma cell line: Good news and bad news. Sci Transl Med 2016; 8: 354re3.

Animals and other research organisms

Policy information about studies involving animals; ARRIVE guidelines recommended for reporting animal research, and Sex and Gender in Research

Laboratory animals	This study involved laboratory animals. Species: Mus musculus. Strain: Athymic NUDE (Janvier, Rj:ATHYM-Foxn1nu/nu) . Gender: female. Age: 7 to 8-week-old. The mice were housed in a barrier facility at an average temperature of 23ºC and humidity of 45-65%. The dark/light cycle was fixed to 12 hours. Experimental and control animals were co-housed.
Wild animals	This study did not involve wild animals.
Reporting on sex	Sex was not considered and it is not known to influence tonic signaling in CAR-T cells. However, we likely have T cells isolated from both male and female donors although we do not know the percentages as the donors were anonymous.
Field-collected samples	This study did not involve the use of field-collected samples
Ethics oversight	All animal experiments were approved by the Northern Stockholm and Uppsala County regional ethics committee (permits N164/15 and 5.8.18-19434/2019), and were performed according to the guidelines for animal experimentation and welfare of Uppsala University.

Note that full information on the approval of the study protocol must also be provided in the manuscript.

Flow Cytometry

Plots

Confirm that:

- [x] The axis labels state the marker and fluorochrome used (e.g. CD4-FITC).
- [x] The axis scales are clearly visible. Include numbers along axes only for bottom left plot of group (a 'group' is an analysis of identical markers).
- [x] All plots are contour plots with outliers or pseudocolor plots.
- [x] A numerical value for number of cells or percentage (with statistics) is provided.

Methodology

Sample preparation	No tissues were extracted for analysis in this study and only cell lines and primary T cells were used for flow cytometry analysis. Cell lines or primary cells were collected and washed in FACS buffer (PBS with 3mM EDTA) prior to staining surface markers for analysis.
Instrument	FACSCanto II; FACS AriaIII (all from BD BioSciences), CytoFLEX (Beckman Coulter).
Software	For flow cytometry: FlowJo v10.8.1, BD FACSDiva v9.0.1, BD FACSDiva 8.0.2, CytExpert 2.4.
Cell population abundance	Cell population abundance i not relevant in this study as more than 95% of the cells are T cells after activation and culture of PBMCs in culture medium supplemented with IL-2.
Gating strategy	Gating strategy is described in Supplementary figure S11-12.<br>Supplementary FIgure S11. Gating strategy to assess proliferation of CAR-Ts (used in Fig. 1I) was performed by gating out the lymphocytes followed by gating out the CAR-Ts (defined as GFP+) out of the single cells. Histogram of division peaks (MFI CellTrace Violet) was subsequently used to determine number of divisions for the CAR-Ts.<br>Supplementary Figure S12. Gating strategy used to identify CAR-Ts (defined as CD3+GFP+ population) in order to assess CAR-T cell size in: Fig. 2H, Fig. 4G, Fig. S5B, Fig. S7F, Fig. S8C and CAR expression level on CAR-Ts in: Fig. 2B, Fig. 4C, Fig. S6A, Fig. S7C. First lymphocytes were gated out followed by single cells. In the single cell population the CAR-Ts were defined (GFP+) within the CD3+ population.

- [x] Tick this box to confirm that a figure exemplifying the gating strategy is provided in the Supplementary Information.
